# ADHD and BPD, two disorders for the same patient? Psychopathological dimensions and other cross-cutting factors in ADHD and BPD: a pragmatic review

**DOI:** 10.1192/j.eurpsy.2024.1363

**Published:** 2024-08-27

**Authors:** M. D. P. Paz-Otero, E. Lozano-Bori, S. Puyal-González, J. Sánchez-Rodríguez, F. Mayor-Sanabria, M. Fernández-Fariña, Í. Alberdi-Páramo

**Affiliations:** ^1^Hospital Universitario Clínico San Carlos, Madrid, Spain

## Abstract

**Introduction:**

The relationship between Borderline Personality Disorder and Attention Deficit Hyperactivity Disorder has been highlighted in different studies over the last few years, with an estimated prevalence of around 15-35% of ADHD in adult patients diagnosed with BPD and a 7.4 times higher risk of developing BPD in patients diagnosed with ADHD.

**Objectives:**

To conduct a pragmatic review of the recent literature on the relationship between ADHD and BPD, so that it serves as a starting point for an in-depth study of the sociodemographic, clinical and cross-sectional dimensional factors of both disorders.

**Methods:**

A bibliographic review of scientific articles published in recent years, in English and Spanish, extracted from the MEDLINE database, which delve into the relationship between BPD and ADHD, will be carried out. In addition, the common psychopathological dimensions, such as impulsivity or emotional dysregulation, as well as the weight of other dimensional factors related to both disorders, will be studied.

**Results:**

The results of the selected articles will be grouped, for a better understanding, in the following sections:

- Clinical factors and shared comorbidities.
- Psychopathological dimensions: impulsivity and emotional dysregulation.
- Other common dimensional factors.

**Conclusions:**

There are common symptoms and etiological or perpetuating factors, as well as comorbidities shared in both conditions, which in many cases make the correct diagnosis and, therefore, the appropriate therapeutic approach to these patients, quite difficult. Taking into account the differential characteristics of BPD and ADHD, it is possible to create different profiles that allow a precise approach to both disorders in those cases in which they coexist in the same patient.

**Disclosure of Interest:**

None Declared

